# Natural-Fiber-Reinforced Polymer Composites for Furniture Applications

**DOI:** 10.3390/polym16223113

**Published:** 2024-11-06

**Authors:** Mariana Ichim, Emil Ioan Muresan, Elena Codau

**Affiliations:** 1 Faculty of Industrial Design and Business Management, “Gheorghe Asachi” Technical University of Iasi, 29 Prof. Dr. Doc. D. Mangeron Blvd, 700050 Iasi, Romania; elena.codau@academic.tuiasi.ro; 2 “Cristofor Simionescu” Faculty of Chemical Engineering and Environmental Protection, “Gheorghe Asachi” Technical University of Iasi, 73 Prof. Dr. Doc. D. Mangeron Blvd, 700050 Iasi, Romania

**Keywords:** composite material, polymeric matrix, natural fiber reinforcement, sustainability, furniture applications

## Abstract

Increasing environmental awareness has driven a shift in furniture production from traditional materials, such as wood and wood-based panels, to sustainable and environmentally friendly alternatives, such as natural-fiber-reinforced (NFR) composites. Environmental consciousness has become a key factor in both production and consumer choices, with growing demand for sustainably sourced materials, eco-friendly manufacturing processes, and durable furniture that helps reduce the impact of disposable products on the environment. This paper analyzes various requirements for natural-fiber-reinforced polymer composites used in furniture applications, including performance, structural–functional, ecological, economical, and safety requirements. It discusses factors influencing the performance of composite materials, such as the selection of matrix and reinforcing fibers, the matrix-to-reinforcement ratio, and the choice of manufacturing technology, as well as the compatibility and bonding between the matrix and fibers. Additionally, several standards commonly used to determine the mechanical, physical, and chemical properties of these materials are presented.

## 1. Introduction

Furniture production has been steadily increasing over the past decade, although it experienced a sharp decline in 2020 due to the pandemic crisis, which resulted in lockdowns, traffic restrictions, material shortages, employee illnesses, and absenteeism [1]. Despite the drop in production, consumer demand for furniture remained high, driven by the increase in employees working from home and the shift to online education, which boosted the need for home office and living space furniture [2].

Within the global furniture industry, the upholstered furniture segment is one of the fastest-developing sectors. According to CSIL, a leading market research company, global consumption of upholstered furniture successfully recovered from the pandemic-induced decline. In 2021, global consumption of upholstered furniture increased by 20% compared to 2020 and by more than 10% when compared to the pre-pandemic year [3].

Some of the challenges that the furniture industry faces are related to the accessibility and pricing of raw materials, sustainability, recycling, and circularity. Wood and wood-based panels constitute the primary raw materials for manufacturing structural furniture elements. The adverse environmental impact of forest overexploitation and rising wood prices has prompted furniture manufacturers to seek alternative materials that are environmentally friendly, readily available, cost-effective, and possess physical and mechanical properties comparable to those of wood. In this regard, research on replacing wood with composite materials has intensified in recent years [4,5,6,7]. Composite materials are made of two or more different materials that form distinct phases, with an interface between them. In composite materials, each constituent retains its individual characteristics, but their combination generates properties different from those of the component materials [8,9]. Replacing wood with composite materials in the structure of furniture products can offer several advantages, such as versatility, sustainability, dimensional stability, cost-effectiveness, and resistance to pests.

Composite materials can be manufactured with specific properties, such as strength, size, and shape, making them more versatile for furniture design and production [10]. They can be easily molded and shaped into various forms, allowing for greater design flexibility and customization options for furniture manufacturers [11,12]. Ciupan et al. reported that replacing wood with composite material reduced the number of reference items from 21 to 2 in the production of a sofa side, which had a beneficial influence on manufacturing costs [13].

Wood is a natural and renewable resource with outstanding functional and aesthetic properties, but its overexploitation has led to environmental harm, prompting many countries to implement regulations on forest use. Composite materials can be obtained from recycled or renewable sources, reducing the demand for natural wood and contributing to sustainability efforts [14,15]. Additionally, composite materials can be designed to be more environmentally friendly by minimizing waste during the manufacturing process [16].

Natural wood can be affected by changes in humidity and temperature, causing it to expand or contract, leading to visual and structural issues in furniture [17,18,19]. Although natural lignocellulosic fibers used as reinforcements in composites are prone to absorbing water, composite materials exhibit better dimensional stability in humid conditions than natural wood and wood-based panels. Several studies have been conducted on wood–polymer composites to improve their dimensional stability, aiming to produce furniture that retains its shape and functionality over time [20,21,22,23].

Since natural reinforcing fibers are easily available, abundant, and affordable, composite materials are cost-effective and can make furniture products more accessible to a wider range of consumers [24,25,26]. Additionally, composite materials can be designed to have consistent properties, reducing the variability and potential defects associated with natural wood, which can also help lower production costs and enhance the quality of finished products [27,28,29].

Wood in its natural state is vulnerable to termites and fungi, which can decrease its durability in furniture applications. In contrast, composite materials can be designed to resist pests like termites, thereby extending the lifespan of furniture products [30].

The aim of this paper is to discuss the requirements for natural-fiber-reinforced polymer composites used in furniture applications, focusing on performance, structural functionality, ecological impact, economic factors, and safety considerations. This paper also presents several standards commonly employed to assess the properties of these materials.

## 2. Considerations Regarding the Requirements for Natural-Fiber-Reinforced Composites Used in Furniture Applications

Furniture is an essential household item, and consumers are often faced with a wide range of choices in terms of shape, style, size, color, and material when purchasing. The process of making furniture has significantly evolved over time, with advances in science and technology leading to a more efficient and faster design process. The selection of appropriate materials during the design phase is vital for ensuring high-quality products and environmentally sustainable manufacturing and disposal processes [31]. The selection of materials is influenced by various factors, such as the desired mechanical properties, processing requirements, cost considerations, and end-use applications. By carefully selecting fibers and combining them with appropriate matrix materials, researchers and engineers can tailor composite materials to meet specific performance needs while also achieving sustainability goals.

Composite materials have been identified as a viable alternative to wood for the structure of furniture. Current trends in composite materials focus on producing environmentally friendly materials that are reinforced with lignocellulosic fibers, as these fibers are renewable and biodegradable and possess the necessary mechanical and physical properties for their intended use.

**Figure 1:**
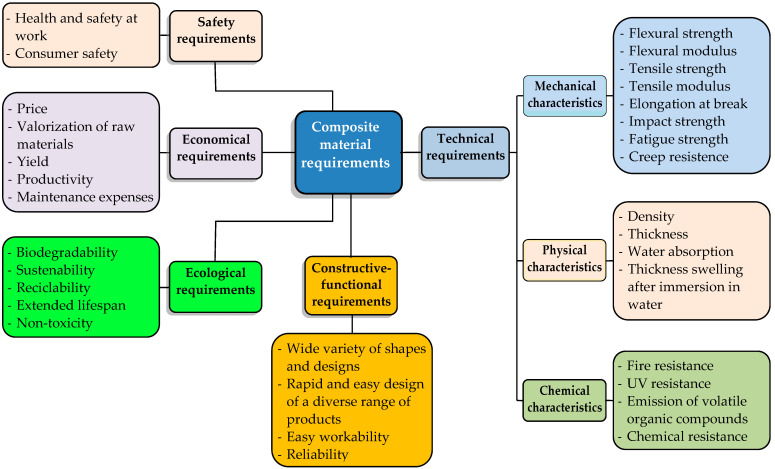
Requirements for NFR composite materials.

The requirements for natural-fiber-reinforced polymer composites used in furniture applications can be grouped into five categories (Figure 1), as follows [32,33]:

- Technical (performance) requirements;
- Constructive–functional requirements;
- Ecological requirements;
- Economical requirements;
- Safety requirements.

The goal of the design process is to maximize the performance of the composite material while adhering to imposed restrictions such as cost, recyclability, sustainability, ease of processing, and consumer safety.

### 2.1. Technical Requirements

The technical requirements for NFR composite materials include the mechanical, physical, and chemical properties these materials must possess to meet their intended purpose.

When designing furniture using composite materials, it is essential to consider a range of performance requirements to ensure that the final product is not only durable and safe but also meets the practical needs of the user. These requirements encompass aspects of structural integrity, aesthetic appeal, environmental resistance, and manufacturing efficiency, all of which are crucial for producing high-quality furniture.

One of the primary considerations is the strength and durability of the composite material. The furniture must possess a sufficient load-bearing capacity to support the intended weight without deforming or failing. This requirement applies to both static loads, such as sitting or placing objects on the furniture, and dynamic loads that involve movement. Additionally, the material must exhibit impact resistance, allowing it to withstand forces without cracking, breaking, or denting. Over time, furniture is subjected to repeated loading and unloading cycles, making fatigue resistance another critical factor in maintaining the material properties and ensuring long-term durability.

In addition to strength, the stiffness and flexibility of the composite material are vital. The furniture needs to be rigid enough to prevent excessive bending or flexing during normal use, ensuring stability and user confidence. The material should possess the ability to return to its original shape after deformation, providing both comfort and longevity.

Dimensional stability is another crucial requirement. The furniture must resist warping, maintaining its shape and dimensions even when exposed to varying environmental conditions such as changes in temperature and humidity. Creep resistance is also important, as the material should not undergo gradual deformation under a continuous load, which could compromise the furniture’s structural integrity over time.

The technical evaluation criteria for NFR composite materials used in furniture products include the following aspects:

- Limit values for resistance to various mechanical stresses, such as tensile strength, bending, impact, fatigue, creep, and abrasion resistance.
- Stiffness and elasticity, measured by the longitudinal and transverse modulus of elasticity.
- Thickness, density, swelling coefficient, and water absorption.
- Resistance to fire, frost, chemicals, and UV radiation, as well as emission of volatile organic compounds.

#### 2.1.1. Mechanical Properties of NFR Composite Materials

The mechanical properties of composite materials determine their response to loads during various manufacturing processes, as well as the long-term performance of finished products. Furniture products made from composite materials must perform reliably (e.g., mechanical failure is undesirable) to remain competitive with similar products made from traditional materials [34]. The inclusion of natural fibers as reinforcements can significantly impact the mechanical behavior of the resulting composite materials and must be carefully evaluated.

The properties of NFR composite materials are influenced by the following factors:

- The type and characteristics of the matrix and the reinforcing fibers (material selection).
- The volumetric/gravimetric ratio between the matrix and the reinforcing fibers.
- The mechanical, physical, and chemical compatibility between the matrix and the reinforcing fibers.
- The type of bonding between the matrix and the reinforcing fibers (physical, mechanical, chemical).
- Manufacturing technology.
- Stacking sequence of plies.

##### 2.1.1.1. Matrix

In the composite material, the matrix serves to embed and bond the fibers together and to transfer the applied load to the fibers. The matrix also redistributes stress when some fibers break.

The polymer matrix can be thermoplastic or thermoset. A thermoplastic polymer becomes soft when heated and hardens when cooled, maintaining its chemical properties through multiple heating and cooling cycles. This stability allows for easy recycling and reprocessing. When heated, thermoplastics can be molded as desired and will retain their shape after solidifying upon cooling to room temperature. Thermoplastic polymers are classified into two groups—amorphous and semi-crystalline—based on their molecular structure. Amorphous polymers have disordered molecular chains and lack a distinct melting temperature, instead melting over a wider range of temperatures. Examples of these polymers include polyvinyl chloride (PVC), polycarbonate (PC), polystyrene (PS), and acrylonitrile–butadiene–styrene (ABS). Semi-crystalline polymers have ordered crystalline regions, where the polymer chains are densely packed and aligned parallel to each other, alternating with disordered amorphous regions. These polymers have a distinct melting temperature, which is the point at which the bonds between the polymer chains break down, causing the material to transition from a solid to a liquid state [35]. Some examples of semi-crystalline polymers are polypropylene (PP), polyethylene (PE), polyethylene terephthalate (PET), polyamide (PA), polyphenylene sulfide (PPS), polyvinylidene fluoride (PVDF), and polyetheretherketone (PEEK). In structural applications, semi-crystalline polymers are used because their crystalline regions provide high toughness, wear resistance, stiffness, and strength [36].

The characteristics of the most commonly used thermoplastic matrices are presented in Table 1 [37,38].

Thermosetting polymers harden permanently when heated, forming three-dimensional crosslinks in the presence of a hardener [39]. After curing, they cannot be remelted, providing them with dimensional stability and resistance to high temperatures. Some examples of thermosets include epoxy, polyester, vinyl ester, and phenolic resins, with their characteristics listed in Table 2 [38].

Compared to thermoplastics, thermosets have the advantage of being stronger and more heat-resistant due to their three-dimensional bonding, but they have the disadvantage of being non-recyclable. Thermoset materials have a limited storage duration. They typically require longer processing times but lower processing temperatures, generally ranging from room temperature to approximately 150 °C.

##### 2.1.1.2. Natural Reinforcing Fibers

In a composite structure, the reinforcing fibers carry the load and provide stiffness and strength to the composite. Due to their intrinsic properties and environmental benefits, natural fibers have gained growing popularity as reinforcements in composite manufacturing, being preferred over synthetic fibers. Natural fibers come from renewable sources and are biodegradable, recyclable, cost-effective, readily available, and lightweight [40,41,42]. They are non-toxic, are non-abrasive to processing equipment, and possess appropriate mechanical properties [43].

**Figure 2:**
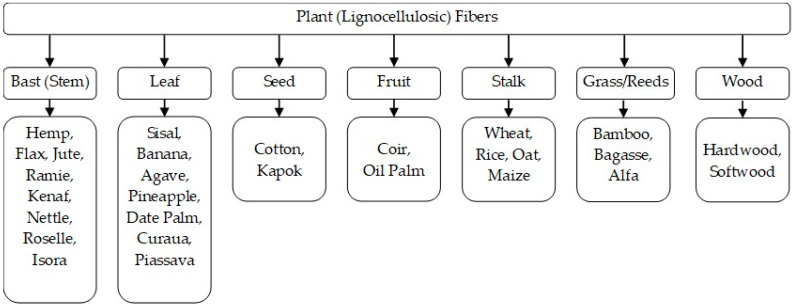
Classification of plant fibers.

Based on their origin, natural fibers can be classified as plant, animal (wool/hair, silk), or mineral fibers (asbestos). Generally, plant fibers are primarily used to reinforce environmentally friendly polymer composites. The classification of plant fibers based on the plant part from which they are extracted is presented in Figure 2 [32,38].

The primary component of plant cell walls is cellulose, the most abundant natural polymer on Earth and a renewable resource. Cellulose fibers aggregate into bundles called microfibrils, which are embedded in a network of hemicellulose and lignin. The content of these three major chemical constituents in the fibers varies widely depending on the plant type. According to Gupta [38], cellulose content varies from 31% in kenaf to 90% in cotton, hemicellulose content ranges from 0.15% in coir to 30% in bamboo, and lignin content ranges between 2% in flax and 45% in coir. Fibers from cereal straw, such as rice, wheat, and oats, generally have a lower cellulose content. In addition to the three major constituents, other components may be present, including pectins, waxes, and ash [44]. The key parameters that define the mechanical properties of plant fibers are the microfibrillar angle (the angle between the direction of cellulose microfibrils and the fiber axis) and the cellulose content [45]. Fibers with higher cellulose contents and lower microfibrillar angles exhibit greater tensile strength and Young’s modulus, whereas higher microfibrillar angles and lower cellulose contents lead to increased ductility in the fibers [46].

The overall properties of plant fibers vary significantly from one plant type to another. Within the same plant type, fiber properties can differ across varieties, between individual plants of the same variety, and even within a single plant. Plant fiber properties are also influenced by cultivation conditions [47,48], timing of the harvest [49], extraction techniques [50,51], treatment [52,53,54,55,56,57], and storage practices [58,59]. Fiber length can range from a few millimeters, as in the case of wood fibers [60], to approximately the length of the plant stem, about 1–3.5 m in the case of jute [61]. Fiber thickness can range from 8 µm in fine cotton [48] to 450 µm in coarse coir fibers [62]. Moreover, in the case of pluricellular technical bast fibers, which are composed of bundles of cells (elementary fibers) held together by the middle lamella (mainly pectin and lignin), variations in thickness occur along the fiber length depending on the degree of division within the technical fiber. The cells are spindle-shaped, with a polygonal cross-section, thick walls, and a central channel, called a lumen. Cell sizes vary between different types of bast fibers and also along the height of the plant stem. Li et al. investigated the variation in hemp cell size along the height of the stem [63]. They found that hemp cells at the base of the stem are shorter and thicker than those at the stem tip. The strength of technical bast fibers is determined by the shape and dimensions of the cells, the number of cells in the bundle, their chemical composition, and the weight of the middle lamella [64]. The more numerous and longer the cells, the more regular their cross-section, and the higher the lignin content, the stronger the technical fibers will be.

The mechanical properties of various plant fibers are given in Table 3 [32,38].

The effect of fiber length on the properties of NFR composites has been investigated by several authors. Anand et al. found that the mechanical properties of silane-treated pineapple leaf fiber-reinforced polyester composites, manufactured using open mold and hand lay-up techniques, tended to improve as fiber length increased up to 20 mm. The experimental range of fiber lengths was between 5 mm and 25 mm [65]. Calotropis gigantea fiber–epoxy composites manufactured by compression molding exhibited the best mechanical properties with a fiber length of 100 mm and a fiber content of 40% [66]. The fiber length varied from 25 mm to 150 mm, and the fiber content ranged from 10% to 50%. For injection molding, a fiber aspect ratio (length/thickness) greater than 10 is considered the minimum required for effective stress transmission [67].

The fiber orientation in the composite material has a significant impact on its properties. Various studies have shown that the best mechanical properties of natural-fiber-reinforced composites are achieved when the fibers are unidirectionally oriented and the applied load is aligned with the fiber orientation [68,69]. In this case, the composites exhibit anisotropic properties. When randomly oriented fibers are used, isotropic composites can be obtained.

The primary disadvantage of using natural fibers as reinforcement in composites is their high moisture absorption, which causes the fibers to swell within the matrix. This swelling weakens the fiber–matrix adhesion, leading to dimensional instability and poor mechanical properties in the composite [70]. Because of the hydrophilicity of natural fibers, water molecules within the microfibrils can vaporize at the high temperatures used in the manufacturing process, leading to the formation of voids in the composite material [71]. Besides the moisture in the reinforcing fibers, other factors that can lead to void formation include the presence of moisture in the resin, volatiles emitted by chemical reactions, and air trapped in the laminate during the layering process [72]. Voids can affect the mechanical properties and reduce the lifespan of the composite [73].

##### 2.1.1.3. Fiber–Matrix Volumetric/Gravimetric Ratio

Adding reinforcing fibers to the polymer matrix aims to fully utilize the high tensile strength of the fibers, resulting in a stronger composite material. If the proportion of reinforcing fibers is too low, the material becomes weaker instead of stronger. When a fiber breaks in the NFR composite structure, the stresses in its surrounding area increase. This elevated stress is transferred to nearby fibers through the matrix. As a result, these neighboring fibers experience increased stress, potentially causing them to break as well. This ongoing process of stress concentration and transfer can ultimately result in the complete failure of the composite [74]. On the other hand, as the reinforcing fiber content increases, the tensile strength of the composite will initially rise until it reaches a threshold. Beyond this point, further increases in fiber content lead to a deterioration in the bonding between the fibers and the matrix, as the fibers come too close together. Consequently, in short-fiber composites, the tensile strength will decrease due to bond failure caused by an excess of fibers.

Using the elastic stress transfer model as the primary theoretical basis, Pan calculated both the maximum and minimum reinforcing fiber volume fractions in composites and demonstrated their dependence on factors such as fiber properties (tensile strength and modulus), fiber aspect ratio (length-to-radius ratio), fiber orientation, matrix properties (Poisson’s ratio and shear modulus), and the fiber–matrix interfacial shear strength [75]. He also noted that incorporating the maximum calculated fiber volume fraction could be challenging, as the amount of reinforcing fiber is constrained by processing technology.

Somashekhar et al. studied the influence of fiber content on the properties of jute-fiber-reinforced PP composites manufactured using a twin-screw extruder [76]. Jute fibers were chopped into 2 mm lengths and treated with a 4% sodium hydroxide solution. The fiber mass content was varied at 10%, 20%, and 30%, while the screw speed was adjusted to 100, 200, and 300 rpm. They found that at a screw speed of 300 rpm, a 10% fiber loading provided better mechanical properties than a 30% fiber loading. Hargitai et al. found that, when using hot pressing manufacturing technology to produce hemp-fiber-reinforced PP composites, the optimal mechanical properties were attained with a hemp fiber mass content of 40–50% [77]. Kazi et al. [78] investigated the effect of fiber percentage (10%, 20%, 30%, and 40%) on the void content of short roselle fiber epoxy composites made using the hand lay-up technique. They observed that the void content increased with higher fiber loadings, ranging from 1.58% for 10% fiber loading to 6.71% for 40% fiber loading. Haddar et al. studied the effect of both fiber loading (0%, 20%, 30%, and 40%) and the maleated polyethylene coupling agent (3%) on the properties of Posidonia oceanica fiber (POF)/high-density polyethylene (HDPE) composites produced by injection molding [79]. The highest stress was observed in the HDPE/POF composite reinforced with 40% POF, which also demonstrated lower strain compared to the composites reinforced with 20% and 30% POF. The incorporation of the coupling agent improved the tensile strength, ductility, impact strength, crystallinity, stabilized torque, and mechanical energy. 

##### 2.1.1.4. Fiber–Matrix Interface

The fiber–matrix interface plays a significant role in determining the performance and durability of composite materials [80]. Improving and optimizing this interface are critical factors in the development and effective use of composite materials [81].

The fiber–matrix interface is the region where the two components of the composite are physically, mechanically, and/or chemically bonded. Due to the poor compatibility between hydrophilic plant fibers and hydrophobic polymer matrices, the interfacial bonding is typically weak, negatively impacting the mechanical properties of the composite materials.

To improve the interfacial bonding between the matrix and reinforcing fibers, and thereby enhance stress transfer across the interface, physical and chemical treatments have been employed [82,83]. Physical treatments of reinforcing fibers, such as plasma and corona treatments [84,85], as well as gamma and UV radiation [86,87], modify the structure and surface properties of the fibers, thereby influencing fiber–matrix adhesion in the composite [88]. Chemical treatments, such as alkaline [89,90,91], acetylation [92], propionylation [93], benzylation [94], silane [95], peroxide [96], permanganate [97], isocyanates [98], and maleated coupling agents treatments [99,100,101], can enhance the interactions at the fiber–matrix interface by creating strong chemical bonds, leading to a substantial improvement in the mechanical performance of the composite.

Research work regarding the influence of various treatments on the properties of composite materials has been reported (Table 4).

##### 2.1.1.5. Fiber–Matrix Interfacial Bonding Mechanisms

In NFR composites, the primary components are the reinforcing fibers and the polymer matrix. The characteristics and behavior of these composites mainly depend on three key factors: the matrix, the reinforcement, and the interface. The interface region governs the stress transfer between the fiber and matrix and is largely influenced by the degree of interfacial adhesion. Adequate interfacial strength ensures that the maximum stress level is effectively sustained across the interface, allowing smooth transfer from the fiber to the matrix. Stress transfer efficiency is influenced by the molecular interactions at the interface, as well as by the thickness and strength of the interfacial zone [88].

The bonding mechanisms that provide adhesion at the fiber–matrix interface include interdiffusion, electrostatic adhesion, chemical reactions, and mechanical interlocking.

The interdiffusion mechanism involves the intertwining of molecules from both the fibers and the matrix. The strength of the interface is determined by several factors, such as the distance over which the molecules are entangled, the degree of molecular entanglement, and the number of molecules per unit area at the interface [108].

Electrostatic adhesion occurs due to the development of opposite electrical charges (cationic and anionic) on the surfaces of the fibers and matrix [109]. Electron transfer takes place between the matrix and fibers, leading to the formation of attractive electrostatic forces at the fiber–matrix interface.

Chemical bonding occurs when chemical bonds (such as hydrogen, covalent, and ionic bonds) form between the fibers and the matrix [110]. To enhance chemical bonding, coupling agents are used. These multifunctional compounds, such as silane and maleated coupling agents, chemically bond with both the fiber and matrix surfaces [111].

Mechanical interlocking takes place when the matrix penetrates the pores or surface irregularities of the fibers. The matrix must evenly fill the cavities on the fiber surface to achieve close and continuous contact, thereby ensuring good bonding quality. The degree of adhesion depends on the fiber porosity, polymer viscosity, and the pressure and duration of the bonding process [112]. Some physical treatments of natural fibers, such as plasma and corona treatments, induce surface etching that enhances surface roughness and leads to a stronger interface with the matrix through mechanical interlocking. Moreover, an increase in mechanical interlocking contributes to the enhancement of other bonding mechanisms [88].

##### 2.1.1.6. Manufacturing Technologies

Due to differences in the chemical structures and properties of thermoset and thermoplastic polymers, the available manufacturing technologies for NFR composites can be categorized based on the type of polymer matrix.

The manufacturing technologies for NFR thermoset composites offer advantages such as easier processing, since the polymer is in a liquid state, along with lower heat and pressure requirements compared to those for NFR thermoplastic composites. However, NFR thermoset composites require a long cure time, and once cured, the thermoset cannot be melted or reshaped, making recycling impossible [113]. Resin transfer molding, vacuum-assisted resin transfer molding, compression molding, and injection molding are some of the most common technologies used for manufacturing NFR thermoset composites. Because plant fibers have low resistance to high temperatures, thermosetting polymers that cure at room temperature can be used without risking thermal degradation of the fibers.

The manufacturing technologies for producing NFR thermoplastic composites are characterized by shorter process cycle times because there are no chemical reactions during curing, leading to a high production rate. Thermoplastic polymers are in a solid state and need to be melted. Nevertheless, thermoplastic composites can be repeatedly melted and solidified, making them easy to recycle at the end of their operational life. The most common manufacturing technologies for thermoplastic-based composites are compression molding, injection molding, and extrusion.

Besides the type of matrix polymer, the selection of manufacturing technology depends on several factors, such as cost, shape, production rate, and fiber orientation. For all manufacturing technologies, the hydrophilic nature of the reinforcing plant fibers poses challenges to the processing conditions. The moisture content of plant fibers typically ranges from 6% to 12%, and therefore it must be reduced to below 3% before processing to minimize negative effects and achieve molded products of good quality. Due to the relatively low degradation temperatures of plant fibers, ranging between 150 °C and 220 °C, the processing temperature must be carefully controlled, especially for thermoplastic-based composites [113]. The processing of NFR composites requires a pressure of approximately 6 MPa to avoid damaging the fibers, even though higher pressure would reduce the void content. Plant fibers with pronounced lumens can be pressed as long as the lumen structure remains intact. Medina showed that when the structure of hemp and kenaf fibers was damaged due to the processing pressure, the mechanical performance of the composite material decreased [114].

Compression molding, injection molding, and extrusion are preferred methods for producing components in furniture products. Compression molding offers advantages such as a high production rate, tight tolerance, and low cost, while injection molding also provides high production rates and precise tolerance, along with the ability to create complex shapes [115]. Extrusion is suitable for high-volume, continuous production of composite sheets or profiles in various shapes.

Compression molding is a technique that uses a closed mold consisting of two halves (a male and a female part) mounted in a hydraulic press to fabricate the composite. The raw material can be in the form of granules, powders, fibers, or liquid resins, but prepregs are commonly used. Prepregs can either be fibrous mats made of reinforcement and matrix fibers or sheet molding compounds (SMCs) containing chopped reinforcement fibers and resin, formulated to allow both fibers and resin to flow under the action of heat and pressure [115]. The precut prepregs are placed inside the preheated mold, and the mold is closed. Heat and pressure are applied, causing the material to flow and conform to the shape of the mold. In the case of a thermoset matrix, heat triggers a chemical reaction that permanently hardens the material, whereas in the case of a thermoplastic matrix, heat melts the polymer, allowing it to flow into the mold before it solidifies upon cooling. The time, heat, and pressure vary depending on the thermal and rheological properties of the matrix, as well as the shape and size of the composites. After curing, the mold is opened, the part is ejected, excess material is trimmed off, and the part undergoes any necessary subsequent processing. Cycle times typically range from two to ten minutes. In a non-isothermal process, the thermoplastic prepreg is preheated in an oven to the processing temperature and then transferred by hand or robot to a preheated mold (20–80 °C). Pressure is rapidly applied while the charge cools upon contact with the mold, a process known as non-isothermal forming. The pressure can be released once the temperature drops below the matrix recrystallization temperature. The cycle time is significantly reduced to approximately 30–60 s [116].

Compression molding can produce large, relatively complex composite parts with a good surface finish and dimensional stability using both thermoset and thermoplastic polymers as matrices. It is also cost-effective for medium to high-volume production batches. Despite the many advantages of compression molding, this process has some limitations: (a) the initial investment for the process is high because of high equipment and mold costs; (b) the process is not suitable for making a small number of parts or for prototyping applications; (c) compression molding of SMC provides nonstructural parts, but by utilizing ribs and stiffeners, structural parts can be manufactured [117].

Zaman used compression molding to produce composites for furniture applications, utilizing treated and untreated coir fibers of 150 mm length and 0.25 mm diameter as reinforcement and PP as the matrix [118]. The fiber content was varied at four levels: 10%, 20%, 30%, and 40%. The coir fibers were first treated with 2% NaOH, followed by 3% silane treatment. The mechanical properties of the treated composites were higher than those of the untreated ones. The tensile strength, tensile modulus, and impact strength increased with the rise in fiber content. The optimum fiber content was found to be 40%. For the treated composite with a 40% fiber load, the tensile strength was around 43 MPa, the tensile modulus approximately 1800 MPa, and the impact strength about 15.7 KJ/m^2^.

Injection molding is a technique in which a liquid fiber-filled polymer is injected under high pressure into a closed, heated mold. The pellets or molding compounds are fed through a funnel-shaped feed hopper into a heated barrel, where they are transported by a rotating screw. As the material passes through the heated barrel, it becomes less viscous and flows more easily. At the end of the barrel, the screw pushes the fiber/polymer mixture through a nozzle into the heated mold, where it solidifies into the shape of the mold. Thermoplastic polymers solidify by cooling, while thermosetting polymers become rigid through crosslinking reactions [117]. Once solidified, the part is ejected by opening the two halves of the mold. In injection molding, the fiber content is limited to a maximum of 30% because a high amount of fibers increases the viscosity of the fiber/polymer mixture, which can cause the nozzle to clog. The length of the reinforcement fibers is short, generally between 1 and 5 mm. Fiber length is restricted due to the excessive friction that may occur as the fibers pass through the nozzle. The mechanical action of the screw can further shorten the fibers, affecting the stress transfer between the matrix and the fibers. Residual stress is a significant concern when working with composites, as it can negatively impact the quality of the final product and often results in premature failure, reducing the product lifespan [119]. These internal stresses develop during the cooling phase, causing compressive residual stresses in the intermediate zones and tensile residual stresses on the surface. Key factors influencing residual stress include (a) different thermal expansion coefficients between the matrix and fibers, (b) the alignment of polymer chains, (c) the formation of a steep pressure gradient, and (d) a non-uniform temperature distribution caused by uneven cooling [32].

Injection molding is primarily used in the thermoplastics industry but has also proven effective in the thermosets industry. It features the shortest cycle time of all molding methods (20–60 s), leading to the highest production rate, which can be further increased by using a multi-cavity mold. While injection molding is commonly applied to create small parts, it is also suitable for producing large, complex structures. Highly automated, this process is ideal for high-volume production, enabling the fabrication of low-cost parts. However, injection molding requires a substantial capital investment and is not suitable for producing prototypes or low-volume parts due to high tooling costs [117].

Koffi et al. studied the mechanical properties of injection-molded HDPE/birch fiber composites. The ground wood fibers had an average length of 0.49 mm and an average diameter of 24.7 µm. A 3% maleic anhydride-grafted polyethylene coupling agent was added to the polymer matrix. The wood fiber content was adjusted to 10%, 20%, 30%, or 40% by weight. Tensile strength and Young’s modulus increased with the rise in fiber content. The composite containing 40% fibers had a tensile strength of 45.5 MPa and a Young’s modulus of 4390 MPa [120].

The extrusion process relies on the application of heat and pressure to force the fiber/polymer mixture through a shaped die, producing long, continuous products with the cross-section required for their application. This method is primarily used for thermoplastic composites. Similar to injection molding, a rotating screw pushes the mixture of fibers and melted polymer through a heated barrel toward a die that determines the cross-sectional shape of the extruded composite. The extrudates are cooled and then cut to the desired size.

Cost-effective and efficient, the extrusion process enables continuous production with high precision and repeatability, reducing additional processing steps while maintaining a consistent quality and lowering production costs. Extrusion is highly effective for producing continuous linear profiles at high production rates, making it well-suited for large-scale manufacturing. Extrusion equipment generally involves lower tooling costs than the complex mold structures required in injection molding. The molds, or dies, used in extrusion are typically simpler to design and manufacture, resulting in shorter setup times and easier operation. However, a primary limitation of extrusion is its inability to create complex, three-dimensional shapes, making it best for products with uniform cross-sections. Additionally, while extrusion can achieve a smooth surface finish, it lacks the range of surface textures and fine details possible with injection molding.

In the furniture industry, the extrusion method is primarily used for producing wood–plastic composites and also serves as a preliminary step to injection molding. When used as a pre-injection stage, extrusion mixes the fibers and matrix into pellets [121]. Shahani et al. recycled plastic waste to produce wood–plastic composites using 40–70% waste wood flour and 25–55% polymer matrix (PP, PE, and PVC) through extrusion molding. Coupling agents, such as 3% silane and 3% maleic anhydride, were used to improve the compatibility between the wood particles and the polymer matrix. Other additives, including colorants, UV stabilizers, antibacterial zinc borate, and fire retardants, were also used. The experimental results demonstrated that an optimal 50% wood–flour proportion yielded a tensile strength of 32.5 MPa, a bending strength of 32.8 MPa, and an impact strength of 17 MPa, while maintaining dimensional stability in humid conditions [122]. Khawale et al. investigated the influence of various compounding parameters of a twin-screw extruder, such as fiber content, fiber feeding position, and barrel temperature, on the properties of sisal/PP composites. The fiber content and barrel temperature were varied at three levels: 20%, 30%, and 40% for fiber content, and 180 °C, 200 °C, and 220 °C for barrel temperature. Additionally, three fiber feeding positions were selected. The fiber length ranged from 2 mm to 4 mm, and the screw speed was set to 50 rpm. Using the Taguchi method, the researchers identified the optimal compounding parameters: 40% fiber content, 180 °C barrel temperature, and a feeding position with three kneading components. The composite produced under these conditions exhibited a tensile strength of 48 MPa and a tensile modulus of 7 GPa. Fiber content and feeding position were the primary factors influencing the tensile properties of the composite material [123].

Several studies have been conducted on the creep and fatigue resistance of NFR composites [124,125]. Creep resistance refers to a composite ability to resist deformation under a constant load over an extended period, while fatigue resistance refers to a composite ability to withstand repeated or cyclic loading without failing. Unlike static loading, which involves a constant load applied to a composite, cyclic loading involves fluctuating stresses that can cause gradual damage over time. Bouafif et al. analyzed the effect of different variables on the creep behavior of wood particles/HDPE composites: particle type (Eastern white cedar, black spruce, and jack pine), size (600–850 µm, 300–425 µm, and 150–300 µm), content (25%, 35%, and 45% wood particle contents), and manufacturing process (extrusion, injection molding, and compression molding) [126]. Ethylene–maleic anhydride copolymer was used as coupling agent (2%). Bürger’s model and the Findley power law were used to model the creep deformation. They found that an increase in wood particle content generally resulted in a decrease in creep resistance. Jack pine composites demonstrated the best creep resistance, likely due to the chemical composition of the fiber surfaces and the effective adhesion mechanism between the wood particles and HDPE. Injection and compression molding processes produced composites with better creep resistance than those made through extrusion, due to differences in their microstructures. Particle size had no significant effect on creep properties. The Findley power law provided a more accurate prediction of the long-term creep behavior of the composites. Stanciu et al. studied the behavior of three types of NFR composites (wood particle/polyester resin, hemp fiber/polyurethane resin, and a mix of flax fabrics and wood particle/polyester resin) subjected to dynamic mechanical analysis [127]. Compared to the hemp/polyurethane composite (HPC), the wood/polyester composite (WPC) and the flax-wood/polyester composite (FWPC) exhibited higher values of storage modulus, indicating a greater capacity to withstand the applied load. An increase in temperature resulted in a reduction in the viscoelastic behavior in all composites. The FWPC samples achieved the highest dynamic modulus values, ranging from 5900 to 6060 MPa. The ability of WPC and HPC samples to store deformation energy gradually decreased as the loading time increased. In the case of WPC and FWPC samples, the loss modulus increased with extended exposure to cyclic loading, whereas the HPC samples showed a decrease in this viscous component. The HPC samples showed the lowest levels of energy dissipation due to internal friction.

##### 2.1.1.7. Stacking Sequence of Plies

In compression molding, usually a stack of several plies is used to feed the mold. The stacking sequence is important because it directly influences the mechanical properties, performance, and behavior of the composite material. Different layers in a composite can be oriented in various directions, optimizing strength and stiffness according to specific load requirements. The stacking sequence determines how the composite handles tensile, compressive, and shear loads in different directions. Ciupan et al. studied the influence of the number and orientation of needle-punched nonwoven plies used to produce composite materials through compression molding. The nonwoven fabrics consisted of 50% hemp fibers, with lengths ranging from 5 to 100 mm and a linear density of 70–80 den, and 50% PP fibers, with lengths ranging from 2 to 60 mm and a linear density of 7–16 den. In the nonwoven fabric, the fibers were randomly oriented. Generally, due to web consolidation by needle punching, the tensile strength of the nonwoven fabric is higher in the machine direction than in the cross-machine direction. Composite materials with 3 and 4 plies were obtained using the following stacking sequences: LLL, LTL, LLLL, and LTLT, where L represents the machine direction and T represents the cross-machine direction. The tensile properties of the composites in the longitudinal, transversal, and diagonal directions were analyzed. They found that the composite material with 4 plies had higher tensile strength due to better homogenization between the hemp and PP fibers compared to the composite material with 3 plies. The best tensile strength was obtained in the longitudinal direction, while the lowest was in the transversal direction, regardless of the stacking sequence. The tensile strength in the diagonal direction fell between the values of the tensile strengths in the longitudinal and transversal directions, except for the stacking sequence LTLT, where it was the highest [128].

The effect of stacking sequence on the properties of composites has been extensively studied, particularly in the case of hybrid composites, which use either a mix of natural fibers [129] or a mix of natural and synthetic fibers as reinforcement [130,131,132].

Various mechanical properties are essential, and standardized methods have been established to evaluate them. Table 5 presents some relevant published standards for mechanical testing of composite materials.

#### 2.1.2. Physical Properties of Composite Materials

The physical properties of NFR composite materials play a crucial role in the design process of furniture products, impacting the choice of composite components, the manufacturing process, and the product performance throughout its operational life. To remain competitive with products made from traditional materials, furniture made from NFR composites must demonstrate an excellent performance. Several physical properties are critically important, and standardized methods have been established to measure them [34].

Density is the mass of a material per unit volume. Composites used in furniture applications require a low density because it results in lighter furniture, making it easier to move, transport, and handle. This is especially important for household furniture that may need to be rearranged frequently or transported during moves. Lightweight furniture also reduces shipping costs and energy consumption in logistics. While being lightweight, NFR composites can offer excellent mechanical properties, enabling the production of strong, durable furniture that does not compromise on performance. In fiber-reinforced polymer composites, density primarily depends on the relative proportions of the reinforcement and matrix, as well as their individual densities. Density plays a crucial role in determining the dimensional stability of the produced composite. Due to the presence of voids within its structure, a low-density composite is capable of retaining more moisture and absorbing more water compared to a high-density composite [157]. The densities of various composite materials are provided in Table 6.

Water absorption in NFR composites can be a significant disadvantage for outdoor and bath furniture. NFR composites absorb water due to the hydrophilic nature and porosity of the fibers, as well as the overall porosity of the composite, the characteristics of the polymer matrix, and capillary action at the fiber–matrix interface [162,163]. Plant fibers have a strong affinity for water [164]. These fibers contain cellulose, hemicellulose, and lignin, which have hydroxyl groups that can form hydrogen bonds with water molecules, leading to water absorption [165]. Additionally, these fibers have a porous structure that allows water to penetrate the fibers, further increasing moisture uptake [166]. During the manufacturing process, voids or microcracks can form within the composite material. These defects can act as pathways for water to enter and spread throughout the composite [167]. Additionally, some polymer matrices used in composites may be somewhat hydrophilic, further contributing to water absorption. The interface between the natural fibers and the polymer matrix is often not perfectly bonded, leaving small gaps or microvoids that can draw in water through capillary action. Water absorption causes the fibers to swell, leading to the formation and expansion of microcracks within the matrix. This weakens the composite mechanical properties and compromises its dimensional stability [168].

It has been discovered that various chemical treatments can reduce the hydrophilic nature of the fibers by decreasing the number of hydroxyl groups, while simultaneously enhancing the bonding between the fibers and the matrix [169]. In a study on the influence of chemical treatments on the water absorption of sisal/polyester composites obtained by resin transfer molding, Sreekumar et al. found that water uptake decreased in the following order: no treatment > heat treatment > silane treatment > permanganate treatment > mercerization > benzoylation. The fiber content was 40%, and the fiber length was 30 mm. All treatments reduced water uptake, with the best results observed in the case of benzoylation, where the presence of the phenyl group enhanced the composite’s hydrophobic nature [170].

The fiber content and dimensions significantly influence the amount of moisture that the NFR composite can absorb [171,172,173]. Jha et al. investigated the water absorption of three types of composites manufactured using the hand lay-up technique: pine cone particle/polycaprolactone (with 0%, 15%, 30%, and 40% filler loading), sisal/epoxy, and jute/epoxy [174]. The latter two composites were produced with different fiber lengths: 5 mm, 10 mm, 15 mm, and 20 mm. Water uptake increased with a higher fiber mass fraction and greater fiber length due to the increase in voids and lignocellulosic content. At saturation, the highest water absorption values for each type of composite were as follows: jute (20 mm)/epoxy 5.49%, sisal (20 mm)/epoxy 3.86%, and pine cone particle (40%)/polycaprolactone 20.96%.

In addition to chemical treatment methods, applying a polymer coating to lignocellulosic fibers [175,176], utilizing coupling agents [177,178,179], and hybridizing natural fibers with synthetic fibers [180,181] have also been recognized as effective techniques for reducing water absorption capacity [181,182]. Wu et al. presented a new technology that improved the water resistance of a hemp/unsaturated polyester composite obtained through the vacuum-assisted resin infusion process [183]. The researchers replaced the traditional vacuum bag with a polyethylene film that adhered in situ to both surfaces of the hemp fiber-reinforced composite. The water absorption of the new composite decreased by 88.5%, 84.8%, and 68.5% after 2 h, 24 h, and 120 h, respectively, compared to the control sample.

In Table 7, some standardized methods for testing the physical properties of composite materials are presented. Several standards related to the melting behavior, specific heat, and thermal conductivity of plastics were added. Understanding the melting behavior is important because it influences processing conditions, material selection, and overall composite performance. Specific heat, which measures the amount of energy needed to increase the temperature of a unit of mass by one degree, is crucial for a product performance during its service life, particularly for outdoor furniture, as it encounters varying environmental temperatures. Thermal conductivity measures how effectively heat flows through a material. Understanding this property is important for composite manufacturing and for the performance of a furniture product exposed to varying environmental temperatures during its service life [34].

#### 2.1.3. Chemical Properties of Composite Materials

The chemical properties of composites, such as fire resistance, ultraviolet (UV) stability, toxicity, and chemical resistance, are largely determined by the nature of the matrix and the reinforcement materials used.

Fire resistance in NFR composites for furniture applications is a vital requirement for ensuring safety, structural integrity, and compliance with regulatory standards. Fire-resistant composites help slow the spread of flames, provide critical time for evacuation, and reduce the risk of fire-related injuries or fatalities [195]. To minimize fire risk and protect both people and property, many countries have adopted strict fire safety standards governing the materials used in furniture manufacturing. For example, the standards ISO 8191-1:1987 and ISO 8191-2:1988 pertain to the assessment of the ignitability of upholstered furniture using a smoldering cigarette and a match-flame equivalent as ignition sources, respectively [196,197]. To fulfill the requirements for furniture applications, the fire resistance of NFR composites must be improved without compromising their essential mechanical properties and thermal performance. Additionally, it is crucial to address environmental considerations [198].

NFR composites exhibit high flammability when exposed to heat flux or flame sources. Improving fire resistance can be achieved through various techniques, such as incorporating additives into the polymer matrix [199,200,201,202], applying flame retardants to the natural fibers [203,204,205,206,207,208,209], building flame-retardant interfacial layers [210], applying flame-retardant coatings on the composite surface [211,212,213], and hybridizing the reinforcing fibers with fire-resistant glass, carbon, or aramid fibers [214,215]. Jeencham et al. studied the effect of three flame retardants (ammonium polyphosphate, magnesium hydroxide, and zinc borate) and their combinations on the mechanical and thermal properties of sisal fiber/PP composites manufactured by injection molding. Maleic anhydride grafted PP was employed as a compatibilizer to improve the compatibility within the system. The flame retardants enhanced the flame resistance of the PP composites and thermal stability without decreasing their mechanical properties. Among the three types tested, ammonium polyphosphate demonstrated the most significant improvement in the flame retardancy of the PP composites [216].

UV stability of composites plays a crucial role in the design and manufacturing of outdoor furniture, which is continuously exposed to various environmental conditions, with sunlight being one of the most significant factors. Composites are favored in outdoor furniture for their strength, durability, and versatility. However, without adequate UV stability, even the most robust composites can deteriorate under prolonged exposure to sunlight. UV radiation can break down the chemical bonds in the polymer matrix of the composite, leading to discoloration, surface cracking, and a loss of mechanical properties [217]. This degradation not only affects the aesthetic appeal of the furniture but also compromises its structural integrity, potentially leading to unsafe conditions for users. By enhancing the UV stability of composites, manufacturers can produce furniture that retains its color, strength, and durability, providing lasting value to consumers.

The most commonly used method to increase the UV resistance of composites is to add UV absorbers, such as benzophenones and benzotriazoles, to the polymer matrix, or to use hindered amine light stabilizers [218,219]. Generally, UV absorbers capture UV radiation and convert it into heat, which is then dissipated through the polymer matrix, while hindered amine light stabilizers inhibit the process of autoxidation [220]. Another method to increase the UV resistance of NFR composites is to apply protective surface coatings that contain UV stabilizers [221]. These coatings form a barrier that shields the underlying composite from UV radiation, reducing the impact of sunlight exposure. However, mechanical deterioration of the coating, such as scratches, can expose the underlying base to UV radiation. Other UV stabilizers used to protect the polymer matrix of the composites from UV degradation include color pigments, such as colcothar, carbon black, and titanium dioxide [222], as well as zinc oxide nanoparticles [223,224], iron oxide nanoparticles [225], titanium dioxide nanoparticles [226], and silica nanopowder [227]. Akindoyo et al. investigated the combined effect of compatibilization and the addition of UV stabilizer on the properties of oil palm empty fruit bunch fiber-reinforced PP composites [228]. The fibers were cut to lengths of 2–5 mm and treated in a 2% NaOH solution. A twin-screw extruder was used to mix the components: 30% fibers, 3% maleic anhydride grafted PP, 2% UV stabilizer, and the matrix, in different combinations—either with a single agent or with both. The composites were then produced using injection molding. The incorporation of the UV stabilizer (CESA-light PPADOL 12,02) slightly decreased the mechanical properties of composites. However, based on discoloration and oxidative induction time analysis, the researchers concluded that the UV stabilizer effectively prevents discoloration and UV degradation in the composites.

The emissions of volatile organic compounds (VOCs), a group of organic chemicals that easily vaporize at room temperature, from furniture are a growing concern. In the context of NFR composites, VOC emissions can originate from the polymer matrix, adhesives, coatings, and other chemical treatments applied during the manufacturing process. Common VOCs mainly include ketones, aldehydes, alcohols, and benzene series [229]. Such compounds may pose health risks and contribute to indoor air pollution [230,231]. The extent of VOC emissions from NFR composite furniture depends on several factors, including the type of polymer used, the presence of chemical additives, and the manufacturing processes. Reducing VOC emissions from NFR composite furniture is a key objective for manufacturers and researchers alike. Efforts are being made to develop low-VOC or VOC-free resins [232,233], as well as sustainable and eco-friendly coupling agents and additives, to minimize their environmental impact [234,235,236,237]. Li et al. studied the VOC emissions and mechanical properties of hemp/PP composites [238]. Because the main source of VOCs is the hemp degumming process, the researchers employed a retting method that involved the natural freezing of hemp stems. The stems were kept in natural conditions, exposed to sunlight, wind, rain, and cold, at temperatures ranging from −10 to −30 °C for 30 to 90 days. The fibers were separated from the woody core through mechanical treatment, then cleaned with a 2% NaOH solution, cut to 70 mm, washed, dried, and treated with 10% urea and 2% KH-550 silane coupling agent. Hemp fibers were mixed with PP fibers in proportions of 40–55%, opened, and the web was consolidated by needle punching into nonwoven fabrics. Compression molding was then used to obtain the composites. Naturally frozen, mechanically treated hemp/PP composites showed lower VOC emissions than untreated ones: 0.092 mg/m^3^ formaldehyde, 0.127 mg/m^3^ acetaldehyde, and no detectable acraldehyde or benzene. The degumming of hemp fibers using the natural freezing–mechanical method reduced the lignin and hemicellulose content, thereby decreasing the source of VOCs. Treatments with urea and the KH-550 silane coupling agent further reduced VOC emissions, imparting the composite with low VOC emission characteristics. The mechanical properties of naturally frozen, mechanically treated hemp/PP composites were superior to those of untreated ones, reaching a peak at a 45% fiber loading, with a tensile strength of 26.93 MPa, a bending strength of 39.26 MPa, and an impact strength of 19.13 kJ/m^2^. Treatments with urea and the KH-550 silane coupling agent further enhanced the mechanical properties.

The chemical resistance of composites used in furniture refers to the ability of these materials to withstand chemical exposures without significant degradation, thereby maintaining the aesthetics and functionality of the furniture pieces. Furniture is frequently subjected to a range of substances, such as household cleaners and disinfectants, which can deteriorate the furniture surface. Additionally, accidental spills of liquids, such as alcohol, vinegar, or oils, can stain the furniture if the composite is not resistant to such substances. The chemical resistance of composites directly affects the aesthetic, maintenance, and service life of furniture pieces, making it an essential factor in material selection and product design. Jawaid et al. investigated the chemical resistance of oil palm empty fruit bunches (EFBs)/woven jute fiber (J_w_)/epoxy hybrid composites manufactured using the hand lay-up method [239]. Two stacking sequences were used: EFB/J_w_/EFB and J_w_/EFB/J_w_, maintaining an EFB/J_w_ weight ratio of 4:1. Additionally, pure EFB and pure J_w_ fiber-reinforced composites were produced. Three solvents (toluene, benzene, and carbon tetrachloride), three acids (acetic acid, hydrochloric acid, and nitric acid), and three alkalis (sodium hydroxide, sodium carbonate, and ammonium hydroxide) were used to evaluate the chemical resistance of the matrix and composites. It was concluded that the hybrid composites were resistant to all the chemicals analyzed.

Several standards that form the basis for testing the burning behavior, UV stability, VOC emissions, and chemical resistance of composites are presented in Table 8.

### 2.2. Constructive–Functional Requirements

A significant constructive–functional requirement for composites in furniture applications is the wide variety of shapes and designs that can be achieved with them. Composites allow for greater flexibility in design, enabling the creation of parts with complex geometries that would typically require numerous individual components if made from conventional materials [13]. Additionally, composite materials can often be molded and processed quickly, which speeds up the production process and allows for a wide variety of design options to be explored efficiently. This flexibility in design not only allows for more innovative and aesthetically pleasing products but also streamlines the manufacturing process by reducing the number of parts needed. The design versatility is one of the key reasons composites are increasingly used in the furniture industry.

Furniture made from composites should be lightweight to be easily moved and assembled, yet strong enough to meet structural demands. A lower density of the composite results in lighter furniture, making it easier to transport, assemble, and rearrange, offering a practical advantage to both manufacturers and consumers.

High reliability is also a notable requirement for products made from composite materials. The inherent properties of these materials contribute to a high probability that the product will consistently perform its intended functions without failure. This reliability is crucial for ensuring that the furniture meets the demands of everyday use, providing durability and longevity that customers can depend on. Along with structural durability, aesthetic durability can be equally important to consumers [253]. The furniture surface should be visually appealing and resistant to scratching, staining, and wear and degradation from regular use.

The ease of assembly and disassembly is a key functional requirement for composite-based furniture. The nature of composite materials often allows for simpler construction methods, making it easier to assemble and disassemble furniture as needed. This feature is especially valuable in today’s market, where modular and easily transportable furniture is in high demand. The combination of these attributes—design versatility, reduced weight, reliability, and ease of assembly—highlights the constructive–functional benefits of using composite materials in furniture production.

### 2.3. Ecological Requirements

Ecodesign requirements address sustainability, recyclability, biodegradability, and the reduction of toxic emissions.

#### 2.3.1. Sustainability

Sustainability refers to the ability of human activities to be conducted in a way that does not deplete available resources or harm the environment, thereby ensuring that future generations can meet their own needs [254].

The development of composite materials to replace traditional wood and wood-based boards in furniture contributes to the responsible use of resources and environmental preservation. Plant fibers used as reinforcing materials are harvested annually and have a higher yield per hectare compared to wood, which has a much longer harvesting cycle of 80–100 years [13]. These plant fibers are renewable, environmentally friendly, and biodegradable, and they can be sourced from agricultural waste or rapidly renewable crops. Replacing wood in furniture structures helps conserve forest resources, reduce pollution, and mitigate the greenhouse effect. Additionally, the production process for NFR composites is often less harmful to the environment due to the reduced use of toxic chemicals. This results in a more sustainable lifecycle for furniture made with NFR composites, minimizing environmental impact and conserving resources for future generations.

#### 2.3.2. Recyclability

Primary resources are becoming increasingly expensive either because they are more difficult to obtain or because their extraction and exploitation have increasingly harmful long-term effects on the environment. In response to these challenges, the European Commission adopted a new Action Plan for the circular economy in March 2020, which outlines measures across the entire lifecycle of products to ensure that resources remain in the EU economy for as long as possible [255].

A report by Eunomia Research & Consulting Ltd. (Brussels, Belgium) revealed that in EU Member States, 10 million tons of furniture are sent to landfills or incinerated annually by consumers and companies. The report also estimated that transitioning to a circular economy in the furniture sector could create 163,000 new jobs, increase the amount of reused or recycled materials by 3.3 to 5.7 million tons, prevent the release of 5.5 million tons of CO_2_, and generate an additional EUR 4.9 billion in gross added value across the EU by 2030 [256].

The design requirements for composite materials aim to ensure the recovery, recycling, and valorization of all waste resulting from the production process, including textile waste in the form of fibers or non-woven material, as well as waste in the form of composite material.

#### 2.3.3. Biodegradability

Biodegradability refers to the gradual breakdown of an organic compound into smaller molecules through microbial action, eventually resulting in the formation of CO_2_, CH_4_, water, or other low-molecular-weight compounds that can no longer be further degraded by microorganisms [257].

**Figure 3:**
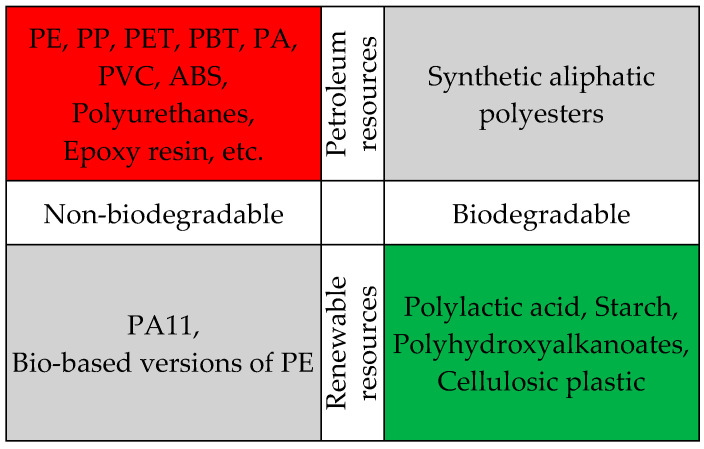
Ecological classification of matrices.

According to Bravo and Vieira [32], the ecological aspects of matrices can be grouped into two categories: their source and their degradability (Figure 3).

Most synthetic polymers, such as PE, PP, PET, PBT, PA, PVC, ABS, etc., are derived from petroleum resources and are not biodegradable. When discarded in the environment, these polymers significantly contribute to pollution. In recent years, increasing attention has been directed toward using natural fibers as reinforcement and biodegradable polymers as matrices in the production of composite materials to reduce their negative environmental impact. Extensive research has been conducted on polylactic-acid-based composites reinforced with natural fibers to evaluate their potential for use in sustainable products [258,259,260,261,262].

Polylactic acid (PLA) is a renewable and biodegradable thermoplastic polymer [263] derived from starch-rich products, such as corn, maize, wheat, rice, potatoes, etc. PLA-based composites exhibit mechanical properties comparable to those of petroleum-based plastics, making them suitable for a wide range of applications, including packaging [264], automotive parts [265], and medical uses [266]. Their versatility is enhanced by their biodegradability, recyclability, high mechanical strength, low toxicity, and ease of processing, allowing for continuous innovation across various industries [267]. In the furniture industry, PLA can be utilized to bind materials currently used in furniture board production, as well as to incorporate waste produced during furniture use or modifications [268].

Table 9 presents some standards that regulate the biodegradation testing of plastic materials.

#### 2.3.4. Toxicity

The main issue with most resins used in the production of wood-derived boards (such as particleboard, fiberboard, medium-density fiberboard, and plywood) is the emission of volatile organic compounds, with formaldehyde being the most prevalent. Formaldehyde is a colorless, flammable gas with a strong, pungent odor and irritating effects on the skin and mucous membranes. In 2004, the International Agency for Research on Cancer classified formaldehyde as carcinogenic to humans [275].

European standards specify requirements for formaldehyde emission levels. The EN 13986:2005+A1:2015 standard classifies wood-based boards by formaldehyde content/emission into two categories: E1 and E2 [276]. Engineered composite materials for furniture applications must comply with the formaldehyde emission class E1. In the case of upholstered furniture, the amount of formaldehyde released from the textile materials is also determined, according to the specifications in the standards ISO 14184-1:2011 and ISO 14184-2:2011 [277,278].

### 2.4. Economical Requirements

Price is a key factor influencing purchasing decisions, both within the value chain and for end consumers. The primary design constraint for composite materials used in furniture products is the need for low production costs.

Production costs can be reduced through several strategies:

- Using inexpensive and readily available raw materials;
- Utilizing plant fiber waste or agro-waste as reinforcement elements [279,280];
- Valorization of agricultural waste into bioplastics [281];
- Using alternative fibers to bast fibers, which are inexpensive and have high yields per hectare, such as willow, poplar, elephant grass, etc. [282,283,284,285];
- Reducing raw material transport costs by sourcing locally;
- Minimizing waste in the manufacturing process;
- Recycling waste materials [16];
- Employing automated, high-productivity production lines;
- Reducing labor costs;
- Lowering maintenance costs for machinery.

In addition to the strategies already mentioned, several other factors can help reduce the cost of composite materials for furniture. One approach is to take advantage of economies of scale, where increasing production volumes lowers the unit cost, as larger batches spread out fixed costs. Investment in research and development can also lead to cost reductions by improving material formulations or manufacturing techniques, such as faster production processes or more efficient curing methods.

Building long-term relationships with raw material suppliers can secure better pricing and reduce fluctuations in material costs.

### 2.5. Safety Requirements

Safety requirements pertain to both the health and safety of employees involved in composite and furniture manufacturing, as well as the safety of the furniture product consumer.

#### 2.5.1. Safety and Health at Work

When designing a new product, the selection of equipment, production methods, and substances must be carried out in a way that ensures the safety and health of employees.

In composite manufacturing, ensuring safety and health at work involves several critical factors. Workers are often exposed to hazardous substances, such as volatile organic compounds, resins, and dust from fiber materials. Proper ventilation, protective clothing, and respiratory equipment are essential to minimize these risks. Additionally, the handling of materials at high temperatures or under pressure can lead to accidents, so proper training and the use of safety protocols are necessary to prevent injuries.

Fire hazards should also be considered, as many materials used in composites are flammable and curing processes may generate heat. Ensuring that proper fire safety measures and equipment are in place is vital.

Regular monitoring, risk assessments, ongoing safety training, and fostering a safety-conscious culture within the workplace can help reduce accidents and long-term health issues in the composite manufacturing process.

#### 2.5.2. Consumer Safety

Products that meet industry standards offer improved quality and safety for consumers. By standardizing product quality and safety, companies build consumer trust and contribute to protecting both the environment and public health. Additionally, providing consumers with clear and accurate information about safety features, care instructions, and potential hazards of furniture helps ensure safe use and maintenance.

In the European Union, the commercialization of products is dependent on the application of the CE marking. This mark indicates that a product complies with specific health, safety, and environmental protection standards, as established by European directives or regulations for each product category [286]. The CE mark is mandatory for goods sold within the European Economic Area, ensuring that they meet essential regulatory requirements.

Product certification plays a pivotal role in fostering trust, facilitating trade, and protecting consumers. Certification provides an objective demonstration of a product’s compliance with regulatory documents and specifications. It not only facilitates market access but also enhances the product’s reputation and global competitiveness.

## 3. Future Prospects

As the demand for sustainable and affordable materials continues to rise, the furniture industry is turning to innovative composites that are both environmentally friendly and economical. Future research and development efforts in this field are focused on sustainable materials, recycled content, energy-efficient production, and sustainable lifecycle management.

One promising direction for environmentally friendly furniture composites is the increased use of bio-based materials for both reinforcement and the matrix. Using a large variety of plant fibers and bio-resins derived from plant oils, algae, or other renewable sources can reduce reliance on petroleum-based resources, leading to furniture materials that are closer to being fully biodegradable at the end of their lifecycle.

Another direction in the research and development of environmentally friendly and economical composites is the incorporation of recycled materials. By using waste products such as sawdust, agricultural residues, textile waste, and recycled plastics, manufacturers can create cost-effective, eco-friendly composites with minimal environmental impact, while also reducing the need for virgin materials.

Traditional composite manufacturing processes are energy-intensive, often involving high-temperature curing cycles. Research into low-energy and low-emission production techniques, such as resin transfer molding at ambient temperatures, could drastically reduce the environmental footprint of composite furniture. The development of room-temperature curing resins can eliminate the need for energy-intensive heating, making the production process more economical and environmentally sustainable.

As 3D printing technology continues to advance, researchers are increasingly investigating its applications and potential advantages within the furniture manufacturing industry. A traditionally time-consuming and costly aspect of the furniture industry is the design process, involving the creation of prototypes, testing of models, and refining of pieces to achieve the final product. Three-dimensional printing simplifies and lowers the cost of this process, allowing designers to produce furniture prototypes rapidly and affordably. Additionally, 3D printing allows for precise, minimal-waste designs that use only the required amount of material. The exploration of 3D printing technology is uncovering new possibilities for design innovation, customization, and efficiency, paving the way for transformative changes in how furniture is conceptualized, prototyped, and produced.

Developing eco-friendly furniture composites also involves considering their entire lifecycle, from production to disposal. Research is increasingly focused on composites that are not only recyclable but also designed for easy disassembly, allowing different materials to be separated and reused. End-of-life solutions are essential for creating a circular economy in the furniture industry, reducing waste, and ensuring that composite materials have a minimal impact on landfills.

## 4. Conclusions

The replacement of wood with natural-fiber-reinforced (NFR) composites in furniture applications presents a growing trend driven by sustainability, performance, and innovation. NFR composites, made from plant-based fibers combined with polymers, offer several advantages over traditional wood. These materials are lightweight, strong, sustainable, versatile, cost-effective, and more dimensionally stable, making them ideal for long-lasting furniture. Composites offer greater design flexibility, allowing the creation of furniture parts with complex geometries.

This review study has provided a thorough examination of the requirements for natural-fiber-reinforced polymer composites in furniture applications, focusing on performance, structural functionality, environmental impact, economic aspects, and safety considerations. It has discussed the factors influencing the performance of composite materials, such as the selection of matrix and reinforcing fibers, the matrix-to-reinforcement ratio, the choice of manufacturing technology, and the compatibility and bonding between the matrix and fibers. Additionally, this paper has outlined several commonly used standards for evaluating the properties of these materials.

NFR composites meet safety and performance standards, making them a viable and eco-friendly substitute for wood. As sustainability becomes a key focus in design and manufacturing, NFR composites are poised to play a major role in shaping the future of furniture production.

## Figures and Tables

**Table 1:** Characteristics of thermoplastics.

Thermoplasts	ρ (g/cm^3^)	E_ten_ (GPa)	σ_tens_ (MPa)	ε_failure_ (%)	T_g_ (°C)	T_m_ (°C)
Polypropylene (PP)	0.91	1.4	31–42	100–600	−20	165
High-density polyethylene (HDPE)	0.96	1.4	26	2–130	−133–−100	130
Polyethylene terephthalate (PET)	1.3	3.5	48–73	30–300	80	250
Polybutylene terephthalate (PBT)	1.3	2.5	56	50–300	40	235
Polyamide 6 * (PA6)	1.1	3.0	35	min 50%	60	220
Polyamide 66 * (PA66)	1.1	1.3	55	min 50%	70	260
Polyamide 12 * (PA12)	1.01	1.1	40	min 50%	40	175
Polyvinyl chloride (PVC)	1.35	3.3	48	145	90	199
Polycarbonate (PC)	1.2	2.2	55–75	80–150	145–150	215–230
Acrylonitrile–butadiene–styrene (ABS)	1.05	3	35		102	105

* As conditioned form.

**Table 2:** Characteristics of thermosets.

Thermosets	ρ (g/cm^3^)	E_ten_ (GPa)	σ_tens_ (MPa)	ε_failure_ (%)	T_g_ (°C)	Cure Shrinkage (%)
Epoxies	1.1–1.4	2.7–4.1	55–130	1–6	170–300	1–5
Phenolics	1.1–1.3	4–7	50–60		175	2–4
Polyesters	1.2–1.5	2.1–3.5	34–105	2	130–160	5–12
Vinyl esters	1.2–1.4	3–3.5	73–81	4–7		5–10

**Table 3:** Mechanical properties of various plant fibers.

Fibers	Density (g/cm^3^)	Elongation (%)	Tensile Strength (MPa)	Young’s Modulus (GPa)
Flax	1.5	2.7–3.2	345–1035	27.6
Hemp	1.5	1.6	690	70
Jute	1.3	1.5–1.8	393–773	26.5
Ramie	1.5	1.2–3.8	400–938	61.4–128
Sisal	1.5	2–2.5	511–635	9.4–22
Coir	1.2	30	175	4–6
Cotton	1.5–1.6	7.0–8.0	287–800	5.5–12.6
Softwood	1.5	–	1000	40
Nettle	–	2.11	1594	87
Curaua	1.4	1.4–4.9	87–1150	11.8–96
Pineapple	1.526	2.4	170–1627	60–82
Bamboo	0.6–1.1	1.3	441	35.9
Piassava	1.4	7.8–21.9	134–143	1.07–4.59
Date palm	-	3.6	135	4.6

**Table 4:** Research work on various treatments of composite components.

Treatment	Composite	Effect of the Treatment	Ref.
Alkaline Acetylation	Bamboo fiber/Recycled HDPE	Both treatments enhanced the tensile properties of the composite materials. The optimal tensile strength of the bamboo/recycled HDPE composite was achieved at concentrations of 2.5% NaOH and 7.5% CH_3_COOH, respectively.	[102]
Alkaline benzoylation	Sugar palm fiber/Epoxy	The fiber treatments increased the tensile stress and tensile modulus of the composites while decreasing the tensile strain. The enhanced bonding strength between the fiber and matrix was due to the removal of the outer layer and impurities from the fiber during chemical treatment.	[103]
Silane	Ijuk fiber/PP	The silane-treated composites exhibited a higher tensile strength and tensile modulus while preserving the elongation at break compared to the untreated composites.	[104]
Maleated PP	Sisal fiber/PP	The composites treated with the 1% maleated PP concentration showed about a 50% increase in tensile strength, a 30% increase in flexural strength, and a 58% increase in impact strength.	[105]
Plasma treatment	Bamboo fiber/Epoxy	The tensile strength of the composite improved after a 30 min plasma treatment with argon, attributed to the removal of lignin and impurities, as well as increased fiber surface roughness.	[106]
Corona treatment	Jute fiber/Polyester	Pullout tests showed that fibers immersed in hot water and then subjected to a 10 min corona discharge treatment exhibited a 34% higher adhesion strength compared to untreated fibers.	[107]

**Table 5:** Standards for mechanical testing of composite materials.

Stress	Standard	Name	Ref.
Tension	ISO 527-1:2019	Determination of tensile properties. Part 1: General principles	[133]
	ISO 527-2:2012	Determination of tensile properties. Part 2: Test conditions for moulding and extrusion plastics	[134]
	ISO 527-3:2018	Determination of tensile properties. Part 3: Test conditions for films and sheets	[135]
	ISO 527-4:2023	Determination of tensile properties. Part 4: Test conditions for isotropic and orthotropic fibre-reinforced plastic composites	[136]
	ISO 527-5:2021	Determination of tensile properties. Part 5: Test conditions for unidirectional fibre-reinforced plastic composites	[137]
Flexure	ISO 14125:1998	Fibre-reinforced plastic composites—Determination of flexural properties	[138]
Impact	ISO 179-1:2023	Determination of Charpy impact properties. Part 1: Non-instrumented impact test	[139]
	ISO 179-2:2020	Determination of Charpy impact properties. Part 2: Instrumented impact test	[140]
	ISO 180:2023	Determination of Izod impact strength	[141]
Compression	ISO 604:2002	Determination of compressive properties	[142]
	ISO 14126:2023	Determination of compressive properties in the in-plane direction	[143]
Fatigue	ISO 13003:2003	Determination of fatigue properties under cyclic loading conditions	[144]
Creep	ISO 899-1:2017	Determination of creep behaviour. Part 1: Tensile creep	[145]
	ISO 899-2:2024	Determination of creep behaviour. Part 2: Flexural creep by three-point loading	[146]
Shear	ISO 15310:1999	Determination of the in-plane shear modulus by the plate twist method	[147]
Hardness	ISO 868:2003	Determination of indentation hardness by means of a durometer (Shore hardness)	[148]
	ISO 2039-1:2001	Determination of hardness—Part 1: Ball indentation method	[149]
	ISO 2039-2:1987	Determination of hardness—Part 2: Rockwell hardness	[150]
General	ISO 291:2008	Standard atmospheres for conditioning and testing	[151]
	ISO 293:2023	Compression moulding of test specimens of thermoplastic materials	[152]
	ISO 1268-1	Methods of producing test plates	[153]
	ISO 294-4:2018	Injection moulding of test specimens of thermoplastic materials. Part 4: Determination of moulding shrinkage	[154]
	ISO 294-5:2017	Injection moulding of test specimens of thermoplastic materials. Part 5: Preparation of standard specimens for investigating anisotropy	[155]
	ISO 295:2004	Compression moulding of test specimens of thermosetting materials	[156]

**Table 6:** Densities of composites.

Composite	Manufacturing Technology	Fiber Content (%)	Density (g/cm^3^)	Reference
Flax/Epoxy	Resin transfer molding	41	1.2804	[158]
Flax/Bio-Epoxy	Hand compression molding	40	1.143	[159]
Ramie/Bio-Epoxy	Hand compression molding	40	1.1259	[159]
Bamboo/Bio-Epoxy	Compression molding	40	1.2497	[160]
Oil palm/Bio-Epoxy	Compression molding	40	1.3038	[160]
Carbon/Epoxy	Compression molding	45	1.4334	[161]

**Table 7:** Standards for testing the physical properties of composite materials.

Property	Standard	Name	Ref.
Density	ISO 1183-1:2019	Methods for determining the density of non-cellular plastics. Part 1: Immersion method, liquid pycnometer method and titration method	[184]
	ISO 1183-2:2019	Methods for determining the density of non-cellular plastics. Part 2: Density gradient column method	[185]
	ISO 1183-3:1999	Methods for determining the density of non-cellular plastics. Part 3: Gas pycnometer method	[186]
Water absorption	ISO 62:2008	Determination of water absorption	[187]
Melting behavior	ISO 3146:2022	Determination of melting behaviour (melting temperature or melting range) of semi-crystalline polymers by capillary tube and polarizing-microscope methods	[188]
	ISO 11357-1:2023	Differential scanning calorimetry (DSC). Part 1: General principles	[189]
	ISO 11357-2:2020	Differential scanning calorimetry (DSC). Part 2: Determination of glass transition temperature and step height	[190]
	ISO 11357-3:2018	Differential scanning calorimetry (DSC). Part 3: Determination of temperature and enthalpy of melting and crystallization	[191]
	ISO 1133-1:2022	Determination of the melt mass-flow rate (MFR) and melt volume-flow rate (MVR) of thermoplastics. Part 1: Standard method	[192]
Specific heat	ISO 11357-4:2021	Differential scanning calorimetry (DSC). Part 4: Determination of specific heat capacity	[193]
Thermal conductivity	ISO 11357-8:2021	Differential scanning calorimetry (DSC)Part 8: Determination of thermal conductivity	[194]

**Table 8:** Standards for testing the chemical properties of composite materials.

Property	Standard	Name	Ref.
Burning behavior	ISO 4589-1:2017	Determination of burning behaviour by oxygen index. Part 1: General requirements	[240]
	ISO 4589-2:2017	Determination of burning behaviour by oxygen index. Part 2: Ambient-temperature test	[241]
	ISO 4589-3:2017	Determination of burning behaviour by oxygen index. Part 3: Elevated-temperature test	[242]
	ISO 5660-1:2015	Heat release, smoke production and mass loss rate. Part 1: Heat release rate (cone calorimeter method) and smoke production rate (dynamic measurement)	[243]
	ISO/TS 5660-3:2012	Heat release, smoke production and mass loss rate. Part 3: Guidance on measurement	[244]
	ISO/TS 5658-1:2006	Spread of flame. Part 1: Guidance on flame spread	[245]
	ISO 5658-4:2001	Spread of flame. Part 4: Intermediate-scale test of vertical spread of flame with vertically oriented specimen	[246]
UV stability	ISO 4892-1:2024	Methods of exposure to laboratory light sources. Part 1: General guidance and requirements	[247]
	ISO 4892-2:2013	Methods of exposure to laboratory light sources. Part 2: Xenon-arc lamps	[248]
	ISO 4892-3:2024	Methods of exposure to laboratory light sources. Part 3: Fluorescent UV lamps	[249]
	ISO 4892-4:2013	Methods of exposure to laboratory light sources. Part 4: Open-flame carbon-arc lamps	[250]
VOCs emission	ISO 16000-9:2024	Indoor air. Part 9: Determination of the emission of volatile organic compounds from samples of building products and furnishing—Emission test chamber method	[251]
Chemical resistance	ISO 175:2010	Methods of test for the determination of the effects of immersion in liquid chemicals	[252]

**Table 9:** Standards for testing the biodegradability of plastic materials.

Standard	Name	Ref.
ISO 10210:2012	Methods for the preparation of samples for biodegradation testing of plastic materials	[269]
ISO 846:2019	Evaluation of the action of microorganisms	[270]
ISO 14853:2016	Determination of the ultimate anaerobic biodegradation of plastic materials in an aqueous system—Method by measurement of biogas production	[271]
ISO 19679:2020	Determination of aerobic biodegradation of non-floating plastic materials in a seawater/sediment interface—Method by analysis of evolved carbon dioxide	[272]
ISO 17088:2021	Organic recycling—Specifications for compostable plastics	[273]
ISO 16929:2021	Determination of the degree of disintegration of plastic materials under defined composting conditions in a pilot-scale test	[274]
